# Supplementary material for: Suppression of an Invasive Native Plant Species by Designed Grassland Communities
Source: Plants (Basel). 2021 Apr 15;10(4):775. doi: 10.3390/plants10040775 (PMC8071313; doi:10.3390/plants10040775)
Supplement: Supplementary file 1 [file plants-10-00775-s001.zip › plants-10-00775-s001.pdf]

Supplementary information to Möhrle et al. "Suppression of an invasive native plant species by designed grassland communities"

Table S1: Species names and traits (and their respective sources) – cutting tolerance, plant height, seed mass, leaf size - and mean germination (see sources at Table S10) used in the experiment.
Table S2: Performance of grassland mixtures in suppressing the native invasive *Jacobaea aquatica* in relation to *J. aquatica* monoculture.
Table S3: Results of a linear mixed-effects model (lmer) for weekly biomass of *Jacobaea aquatica* with corrected outliers and rescaled data. Wald tests (type 3) with community type, traits constrained and diversified (and the respective interactions) as well as with phylogenetic diversity (PD) and CWM values of plant height, leaf area and seed mass type as predictors were applied to test for significant differences.
Table S4: Tukey test results for differences in weekly *Jacobaea aquatica* biomass between base communities. Comparisons of wet against mesic communities within the different experimental periods.
Table S5: Results of a HSD test following an ANOVA for the effects of the treatments within mesic communities on weekly biomass *Jacobaea aquatica*.
Table S6: Results of a HSD test following an ANOVA for the effects of the treatments within wet communities on weekly biomass *Jacobaea aquatica*.
Table S7: Twelve experimental plant community mixtures with constrained and diversified traits and corresponding seed mixture codes, as well as a reference grassland mixture produced by Bavarian State Research Centre for Agriculture (LfL) and a *Jacobaea aquatica* monoculture.
Table S8: Experimental settings of the experiment in a greenhouse of the Centre of Greenhouses and Laboratories Dürnast, Technical University of Munich, Germany
Table S9: Mean weekly biomass per tray (g 1.5 dm^{-2}). Values represent average and std. error for the main community types.
Table S10: Literature sources and own germination tests data for the species used in the experiment.
Figure S1: Proportional native to invader biomass in the second (left) and third (right) experimental periods.

Table 1. Species names and traits (and their respective sources) – cutting tolerance, plant height, seed mass, leaf size, and mean germination (see sources at Table S10) used in the community experiment on suppression of an invasive native plant (Jacobaea aquatica).

Species	Cutting tolerance (BiolFlor)	Plant height (m) (LEDA)	Seed mass (mg) (LEDA)	Leaf size (mm^2) (LEDA)	Mean germination (%)
Achillea millefolium	7	0.40	0.13	411.0	93.2
Anthoxanthum odoratum	7	0.17	0.63	460.0	80.0
Arrhenatherum elatius	6	1.28	3.08	1223.0	75.0
Caltha palustris	4	0.26	1.03	19640.0	86.0
Carex flacca	4	0.28	1.02	634.7	75.0
Carum carvi	6	0.45	1.92	1254.9	60.0
Centaurea jacea	5	0.85	1.99	708.0	64.6
Cynosurus cristatus	7	0.55	0.55	347.3	75.0
Dactylis glomerata	8	0.45	0.91	1449.0	83.0
Festuca pratensis	6	0.55	1.95	527.5	90.0
Festuca rubra	9	0.48	0.91	382.0	85.0
Filipendula ulmaria	3	0.79	0.75	10234.5	86.0
Galium palustre	4	0.44	0.91	32.5	43.7
Lathyrus pratensis	5	0.57	12.52	158.3	29.0
Leontodon hispidus	5	0.24	1.11	2280.6	83.0
Leucanthemum vulgare	6	0.36	0.38	669.6	70.5
Lolium perenne	8	0.13	1.98	402.0	85.0
Lotus corniculatus	6	0.43	1.40	132.0	63.3
Lythrum salicaria	3	0.71	0.09	1742.3	90.0
Medicago lupulina	7	0.25	1.78	509.3	29.0
Mentha aquatica	4	0.38	0.13	1722.4	86.0
Molinia caerulea	3	0.79	0.71	1286.6	64.0
Myosotis scorpioides	5	0.28	0.30	1086.0	43.0
Phleum pratense	8	0.37	0.59	380.0	50.0
Pimpinella major	5	0.66	2.07	9334.0	60.0
Pimpinella saxifraga	6	0.41	1.15	2609.5	30.0
Plantago lanceolata	7	0.16	1.62	2567.0	46.5
Plantago media	4	0.37	0.27	3968.0	65.0
Poa pratensis	9	0.30	0.27	1025.0	85.0
Potentilla erecta	3	0.17	0.48	148.5	42.0
Prunella vulgaris	9	0.12	0.69	675.3	32.5
Jacobaea aquatica	5	0.32	0.38	3784.0	14.2
Silene vulgaris	4	0.27	0.94	1179.5	98.0
Succisa pratensis	3	0.34	1.37	4860.6	84.0
Trifolium pratense	7	0.28	1.58	715.5	70.0
Trifolium repens	7	0.35	0.59	682.0	56.0
Trisetum flavescens	7	0.55	0.30	385.0	47.0

Table S1: Performance of grassland mixtures in suppressing the native invasive Jacobaea aquatica *in relation to* J. aquatica *monoculture.*

**Grassland mixture**	**Mean weekly *J. aquatica* biomass in g**	**Proportion to weekly *J. aquatica* monoculture biomass**	**Treatment decrease to *J. aquatica* monoculture**
LfL	0.03	0.06	0.94
M-La*Ph*	0.11	0.23	0.77
M-La*Sm*	0.07	0.14	0.86
M-Ph*La*	0.14	0.30	0.70
M-Ph*Sm*	**0.05**	**0.11**	**0.89**
M-Sm*La*	**0.16**	**0.36**	**0.64**
M-Sm*Ph*	0.10	0.22	0.78
W-La*Ph*	0.10	0.22	0.78
W-La*Sm*	0.10	0.23	0.77
W-Ph*La*	0.08	0.18	0.82
W-Ph*Sm*	0.08	0.17	0.83
W-Sm*La*	0.10	0.23	0.77
W-Sm*Ph*	0.13	0.28	0.72
Monoculture *J. aquatica*	0.46	1.00	

Table S2: Results of a linear mixed-effects model (lmer) for weekly biomass of Jacobaea aquatica *with corrected outliers and rescaled data. Wald tests (type 3) with community type, traits constrained and diversified (and the respective interactions) as well as with phylogenetic diversity (PD) and CWM values of plant height, leaf area, and seed mass type as predictors were applied to test for significant differences.*

**Model with community type and trait constrained/diversified**			
	**Chisq**	**Df**	**Pr(>Chisq)**
(Intercept)	0.19	1	0.66
community_type	0.03	1	0.87
trait_constrained	1.39	2	0.50
trait_diversified	24.08	2	5.90E-06
community_type:trait_constrained	0.41	2	0.81
community_type:trait_diversified	14.67	2	0.0006

**Model with interaction of community type and trait constrained/diversified**			
	**Chisq**	**Df**	**Pr(>Chisq)**
(Intercept)	0.04	1	0.83
community_type	0.18	1	0.67
trait_constrained	0.39	2	0.82
trait_diversified	16.99	2	0.0002
community_type:trait_constrained	1.00	2	0.60
community_type:trait_diversified	8.53	2	0.01
trait_constrained:trait_diversified	0.16	1	0.69
community_type:trait_constrained:trait_diversified	0.59	1	0.44

**Trait model**			
	**Chisq**	**Df**	**Pr(>Chisq)**
(Intercept)	0	1	1
Leaf area	4.21	1	0.040
Plant height	0.27	1	0.60
Seed mass	10.95	1	0.0009
PD	5.34	1	0.020
Weekly native biomass	16.00	1	6.31E-05

Table S3: Tukey test results for differences in weekly Jacobaea aquatica *biomass between base communities. Comparisons of wet against mesic communities within the different experimental periods.*

**Study periods**	**Diff**	**lwr**	**upr**	**p adj**	**Mean weekly *J. aquatica* biomass (g)**	
					Wet communities	Mesic communities
Period 1	0.030	-0.007	0.067	0.108	0.18	0.15
Period 2	0.005	-0.020	0.029	0.662	0.05	0.05
Period 3	-0.051	-0.090	-0.012	0.011	0.06	0.10

Table S4: Results of a HSD test following an ANOVA for the effects of the treatments within mesic communities on weekly biomass Jacobaea aquatica.

**HSD Test for weekly *J. aquatica* biomass in MESIC community, treatments, means**					
	**Weekly_sbm**	**Std**	**r**	**Min**	**Max**
**M-ChLa**	0.138	0.110	18	0.006	0.415
**M-ChSm**	0.051	0.056	18	0.005	0.204
**M-LaCh**	0.106	0.090	18	0.001	0.299
**M-LaSm**	0.065	0.058	18	0	0.193
**M-SmCh**	0.101	0.078	18	0.005	0.243
**M-SmLa**	0.163	0.081	18	0.031	0.295
**Mean Square Error**					0.004
**DF Error**					100
**Alpha:**					0.05
**Critical Value of Studentized Range**					4.11
**Minimum Significant Difference**					0.067

Table S5: Results of a HSD test following an ANOVA for the effects of the treatments within wet communities on weekly biomass Jacobaea aquatica.

HSD Test for weekly *J. aquatica* biomass in WET community, treatments, means					
	**Weekly_sbm**	**Std**	**r**	**Min**	**Max**
W-Ph*La*	0.080	0.078	18	0	0.245
W-Ph*Sm*	0.078	0.063	18	0.006	0.185
W-La*Ph*	0.100	0.108	18	0	0.366
W-La*Sm*	0.104	0.077	18	0.019	0.281
W-Sm*Ph*	0.126	0.087	18	0.007	0.346
W-Sm*La*	0.104	0.108	18	0.005	0.377
Mean Square Error					0.004
DF Error					100
Alpha					0.05
Critical Value of Studentized Range					4.11
Minimum Significant Difference					0.063

Table S6: Twelve experimental plant communities mixtures with constrained and diversified traits and corresponding seed mixture codes, as well as a reference grassland mixture produced by Bavarian State Research Centre for Agriculture (LfL) and a Jacobaea aquatica *monoculture.*

**Community type**	**Trait constrained**	**Trait diversified**	**Abbreviation**
Mesic grassland	Plant height	Leaf area	M-Ph*La*
	Plant height	Seed mass	M-Ph*Sm*
	Leaf area	Plant height	M-La*Ph*
	Leaf area	Seed mass	M-La*Sm*
	Seed mass	Plant height	M-Sm*Ph*
	Seed mass	Leaf area	M-Sm*La*
Wet grassland	Plant height	Leaf area	W-Ph*La*
	Plant height	Seed mass	W-Ph*Sm*
	Leaf area	Plant height	W-La*Ph*
	Leaf area	Seed mass	W-La*Sm*
	Seed mass	Plant height	W-Sm*Ph*
	Seed mass	Leaf area	W-Sm*La*
Reference grassland			LfL
Invasive monoculture			*J. aquatica*

Table S7: Experimental settings of the experiment in a greenhouse of the Centre of Greenhouses and Laboratories Dürnast, Technical University of Munich, Germany

	**Period 1**	**Period 2**	**Period 3**
**Timespan**	May–July 2019 (10 weeks)	July–Nov. 2019 (15 weeks)	Nov. 2019–April 2020 (22 weeks)
**Fertilisation**	Substrate fertilizer NPK 14-10-18 0.5 kg/ m^3	From 07.10 on: Universol Blue (18-11-18 + 2.5 MgO + TE) through irrigation water	Universol Blue (18-11-18 + 2.5 MgO + TE) through irrigation water
**Irrigation**	Flooding 2x a day for 30 min (morning and afternoon)	Flooding every 2nd day for 30 min (morning and afternoon)	Flooding every 2nd day for 30 min (morning and afternoon)

Table S8: Mean weekly biomass per tray. Values represent average and std. error for the main community types.

Community type	Mean weekly biomass per tray (g 1.5 dm^{-2})	Std. error
LfL reference	2.25	0.39
Mesic community	1.34	0.15
J. aquatica monoculture	0.46	0.11
Wet community	1.33	0.20

Table S9: Literature sources and own germination tests data for the species used in the experiment; germination rates were mean germination of sources

Species	Source
Achillea millefolium	Germination test performed by Sandra Rojas Botero, Chair of Restauration Ecology TUM; personnal communication
Anthoxanthum odoratum	Harris, G. S. 1961. The periodicity of germination in some grass species. *New Zealand Journal of Agricultural Research* 4:253-260. Williams, E. D. 1983a. Effects of temperature, light, nitrate and pre-chilling on seed germination of grassland plants. *Annals of Applied Biology* 103:161-172. https://wiki.bugwood.org/Anthoxanthum_odoratum#cite_note-harris-19
Arrhenatherum elatius	Tanphiphat, K. 1990. Biology and control of tuber oatgrass (*Arrhenatherium elatius* (L.). Resl. Var. *bulbosum* (Wild.) Spenn.). PhD Thesis. Oregon State Univ. 107 pp.
Caltha palustris	Grime, J. P., Mason, G., Curtis, A. V., Rodman, J., Band, S. R. 1981. A comparative study of germination characteristics in a local flora. *Journal of Ecology* 69:1017-059.
Carex flacca	Schütz, W., Rave, G. 1999. The effect of cold stratification and light on the seed germination of temperate sedges (*Carex*) from various habitats and implications for regenerative strategies. *Plant Ecology* 144: 215-30. Accessed February 23, 2021. http://www.jstor.org/stable/20050829.
Carum carvi	Galambosi, B., Peura, P. 1996. Agrobotanical features and oil content of wild and cultivated forms of caraway (*Carum carvi* L.). *Journal of Essential Oil Research* 8:389-397, DOI: 10.1080/10412905.1996.9700646
Centaurea jacea	Germination test performed by Sandra Rojas Botero, Chair of Restauration Ecology TUM; personnal communication
Cynosurus cristatus	Harris, G. S. 1961. The periodicity of germination in some grass species. *New Zealand Journal of Agricultural Research* 4:253-260.
Dactylis glomerata	Harris, G. S. 1961. The periodicity of germination in some grass species. *New Zealand Journal of Agricultural Research* 4:253-260.
Festuca pratensis	Żurek, G. 1999. Effect of seed storage on germplasm integrity of meadow fescue (*Festuca pratensis* Huds.). *Genetic Resources and Crop Evolution* 46:485-490. https://doi.org/10.1023/A:1008750501273
Festuca rubra	Harris, G. S. 1961. The periodicity of germination in some grass species. *New Zealand Journal of Agricultural Research* 4:253-260.
Filipendula ulmaria	Patzelt, A., Wild, U., Pfadenhauer, J. 2001. Restoration of wet fen meadows by topsoil removal: Vegetation development and germination biology of fen species. Res*toration Ecology* 9:127-136. https://doi.org/10.1046/j.1526-100x.2001.009002127.x
Galium palustre	Kathrin Möhrle (*G. album*); Ludewig, K., Zelle, B., Eckstein, R. L., Mosner, E., Otte, A., Donath, T. W. 2014. Differential effects of reduced water potential on the germination of floodplain grassland species indicative of wet and dry habitats. *Seed Science Research* 24:49-61. 10.1017/S096025851300038X.
Lathyrus pratensis	Germination test performed by Sandra Rojas Botero, Chair of Restauration Ecology TUM; personnal communication
Leontodon hispidus	Tofts, R., Silvertown, J. 2002. Community assembly from the local species pool: an experimental study using congeneric species pairs. *Journal of Ecology* 90:385-393. https://doi.org/10.1046/j.1365-2745.2001.00673.x
Leucanthemum vulgare	Germination test performed by Kathrin Möhrle, Chair of Restauration Ecology TUM; personnal observation.
Lolium perenne	Williams, E. D. 1983. Effects of temperature, light, nitrate and pre chilling on seed germination of grassland plants. *Annals of Applied Biology* 103:161-172. https://doi.org/10.1111/j.1744-7348.1983.tb02752.x
Lotus corniculatus	Germination test performed by Sandra Rojas Botero, Kathrin Möhrle, Chair of Restauration Ecology TUM; personnal communications
Lythrum salicaria	Patzelt, A., Wild, U., Pfadenhauer, J. 2001. Restoration of wet fen meadows by topsoil removal: Vegetation development and germination biology of fen species. Res*toration Ecology* 9:127-136. https://doi.org/10.1046/j.1526-100x.2001.009002127.x
Medicago lupulina	Sharpe, S., Boyd, N. 2019. Black medic (*Medicago lupulina*) germination response to temperature and osmotic potential, and a novel growing degree-day accounting restriction for heat-limited germination. *Weed Science* 67:246-252. doi:10.1017/wsc.2018.68; Boe, A., Bortnem, R., Johnson, P. J. 2017. Changes in weight and germination ability of black medic seed over a growing season, with a new seed predator. *Proceedings of the South Dakota Academy of Science* 95:105.

Mentha aquatica	Grime, J. P., Mason, G., Curtis, A. V., Rodman, J., Band, S. R. 1981. A comparative study of germination characteristics in a local flora. *Journal of Ecology* 69:1017-1059.
Molinia caerulea	Grime, J. P., Mason, G., Curtis, A. V., Rodman, J., Band, S. R. 1981. A comparative study of germination characteristics in a local flora. *Journal of Ecology* 69:1017-1059.
Myosotis scorpioides	Lenssen, J. P. M., ten Dolle, G. E., Blom, C. W. P. M. 1998. The effect of flooding on the recruitment of reed marsh and tall forb plant species. *Plant Ecology* 139:13-23 (1998). https://doi.org/10.1023/A:1009754505028
Phleum pratense	Grime, J. P., Mason, G., Curtis, A. V., Rodman, J., Band, S. R. 1981. A comparative study of germination characteristics in a local flora. *Journal of Ecology* 69:1017-1059.
Pimpinella major	Grime, J. P., Mason, G., Curtis, A. V., Rodman, J., Band, S. R. 1981. A comparative study of germination characteristics in a local flora. *Journal of Ecology* 69:1017-1059.
Pimpinella saxifraga	Germination test performed by Kathrin Möhrle, Chair of Restauration Ecology TUM; personnal observation
Plantago lanceolata	Germination test performed by Kathrin Möhrle, Chair of Restauration Ecology TUM; personnal observation
Plantago media	Cojocariu, L., Horablaga, N. M., Horablaga, A., Cojocariu, A., Bordean, D. M., Borozan, A., … Rujan, C. 2013. *Plantago media* L. germination response. *Research Journal of Agricultural Science* 45:58-63.
Poa pratensis	Pill, W., Korengel, T. 1997. Seed priming advances the germination of Kentucky Bluegrass (*Poa pratensis* L.). *Journal of Turfgrass Management* 2. 10.1300/J099v02n01_03.
Potentilla erecta	Lopez Del Egido, L., Toorop, P. E., Lanfermeijer, F. C. 2019. Seed enhancing treatments: comparative analysis of germination characteristics of 23 key herbaceous species used in European restoration programmes. *Plant Biology* 21:398-408. doi: 10.1111/plb.12937. Epub 2018 Dec 10. PMID: 30427114.
Prunella vulgaris	Germination test performed by Kathrin Möhrle, Chair of Restauration Ecology TUM; personnal observation
Jacobea aquatica	Germination test performed by Hugo E. Reyes Aldana, personnal communication
Silene vulgaris	Partzsch, M. 2011. Zur Keimungsbiologie ausgewählter Xerothermrasenarten -Teil 2: Caryophyllaceae. *Hercynia* 44:127-144.
Succisa pratensis	Patzelt, A., Wild, U., Pfadenhauer, J. 2001. Restoration of wet fen meadows by topsoil removal: Vegetation development and germination biology of fen species. Res*toration Ecology* 9:127-136. https://doi.org/10.1046/j.1526-100x.2001.009002127.x
Trifolium pratense	Germination test performed by Kathrin Möhrle, Chair of Restauration Ecology TUM; personnal observation
Trifolium repens	Baig, Z., Shaukat, S., Ahmed, M., Khan, A. 2015. Effect of salninty on germination of *Trifolium repens, Dactylis glomerate* and *Medicago sativa*. *Journal of Biology* 5:107-113.
Trisetum flavecens	Dixon, J. 1995. *Trisetum flavescens* (L.) Beauv. (T. pratense Pers., Avena flavescens L.). *Journal of Ecology* 83:895-909. doi:10.2307/2261427

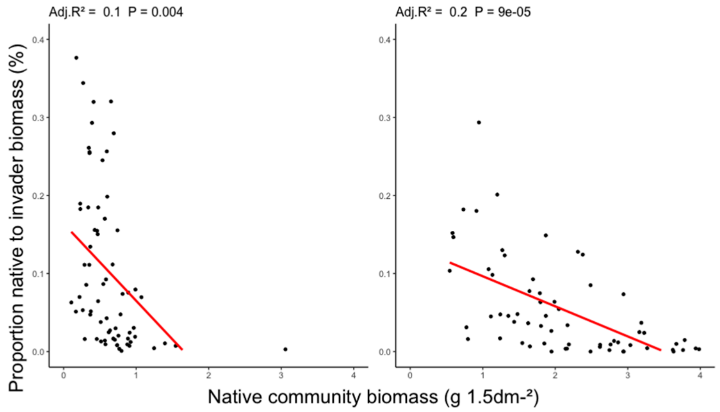

Figure S1: Proportional native to invader biomass in the second (left) and third (right) experimental periods.
